# Supplementary material for: MEME-LaB: motif analysis in clusters
Source: Bioinformatics. 2013 May 14;29(13):1696–7. doi: 10.1093/bioinformatics/btt248 (PMC3694638; doi:10.1093/bioinformatics/btt248)
Supplement: Supplementary Data [file supp_btt248_Supplementary_Data.zip › Supplementary_Data/Results_Files/Results_Files_Readme.rtf]

These files are provided as an example of the output generated by the MEME-LaB tool. It is this result from which figure 1 is taken.
The full result file is obtained by opening results.html.
